# TRPM8 is a neuronal osmosensor that regulates eye blinking in mice

**DOI:** 10.1038/ncomms8150

**Published:** 2015-05-22

**Authors:** Talisia Quallo, Nisha Vastani, Elisabeth Horridge, Clive Gentry, Andres Parra, Sian Moss, Felix Viana, Carlos Belmonte, David A. Andersson, Stuart Bevan

**Affiliations:** 1 Wolfson Centre for Age Related Diseases, King's College London, London SE1 1UL, UK; 2 Instituto de Neurociencias de Alicante, Universidad Miguel Hernández-CSIC, San Juan de Alicante E-03550, Spain

## Abstract

Specific peripheral sensory neurons respond to increases in extracellular osmolality but the mechanism responsible for excitation is unknown. Here we show that small increases in osmolality excite isolated mouse dorsal root ganglion (DRG) and trigeminal ganglion (TG) neurons expressing the cold-sensitive TRPM8 channel (transient receptor potential channel, subfamily M, member 8). Hyperosmotic responses were abolished by TRPM8 antagonists, and were absent in DRG and TG neurons isolated from *Trpm8*^*−/−*^ mice. Heterologously expressed TRPM8 was activated by increased osmolality around physiological levels and inhibited by reduced osmolality. Electrophysiological studies in a mouse corneal preparation demonstrated that osmolality regulated the electrical activity of TRPM8-expressing corneal afferent neurons. Finally, the frequency of eye blinks was reduced in *Trpm8*^*−/−*^ compared with wild-type mice and topical administration of a TRPM8 antagonist reduced blinking in wild-type mice. Our findings identify TRPM8 as a peripheral osmosensor responsible for the regulation of normal eye-blinking in mice.

## Abstract (web summary)

Specific peripheral sensory neurons respond to increases in extracellular osmolality but the mechanism responsible for the neuronal excitation is unknown. Here the authors show that the cold-activated ion channel, TRPM8, also acts as an osmosensor *in vivo*, in cultured mouse sensory neurons and when heterologously expressed in cells.

## Graphical abstract

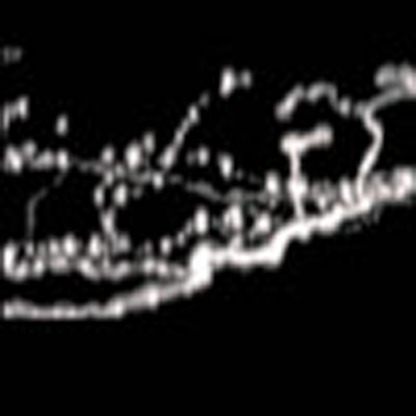

Peripheral sensory nerves innervating the skin and internal organs convey information about the external and internal environment. Individual neurons display sensitivities to one or more modalities and together they are responsible for sensing various thermal, chemical and mechanical stimuli. Among these stimuli, an increase or decrease in extracellular osmolality can excite mammalian sensory neurons innervating various organs and tissues including the airways, gastrointestinal tract, liver and cornea^1^,^2^,^3^,^4^,^5^,^6^. The mechanisms responsible for excitation are thought to involve either a mechanical perturbation of the membrane elicited by changes in cell volume or osmotic activation of intracellular pathways^7^,^8^. Transient receptor potential (TRP) channels play important roles in the transduction of thermal, mechanical and chemical stimuli^9^ and have been implicated in the responses to osmotic stimuli in various cell types^10^. Studies in invertebrates provided evidence that TRP channels can contribute to sensitivity to hyperosmotic stimuli in specialized neurons. Notably, a transient receptor potential vanilloid (TRPV) family orthologue, osm-9, is required for responses to hyperosmotic solutions in *C. elegans*^11^ and the TRP channels *water witch* and *nanchung* are required for the ability of *D. melanogaster* to detect environmental humidity^12^. In mammals, an N-terminal variant of TRPV1 is required for sensitivity to hyper-osmotic stimuli in magnocellular secretory neurons in the central nervous system^13^ and TRPV4 has been reported to mediate peripheral sensory neuron responses to hypo-osmotic solutions^2^,^7^.

The identities of sensory neurons that respond to physiologically relevant increases in osmolality and the underlying molecular mechanism have not been established. Here we used changes in [Ca^2+^]_i_ and electrophysiological recordings from isolated dorsal root ganglion (DRG) and trigeminal ganglion (TG) neurons to show that TRPM8 is required for hyperosmotic responses in neurons and sensory terminals. We also show that expression of TRPM8 confers cellular sensitivity to small changes in osmolality, which modulate the temperature sensitivity of TRPM8. Increasing osmolality evokes depolarization and action potential firing in TRPM8-expressing sensory neurons, whereas inhibition of TRPM8 evokes hyperpolarization. Furthermore, we show that TRPM8 acts as an osmotic sensor in the cornea. Here hyperosmotic solutions increase and hypo-osmotic solution decrease nerve terminal impulse (NTI) activity and osmotic activation of TRPM8 provides a peripheral neuronal drive that maintains normal eye blinking. These results demonstrate that TRPM8 acts as a multimodal sensor of thermal and osmotic stimuli and identify a new role for TRPM8 in the eye.

## Results

### Sensory neuron responses to hyperosmotic stimuli

**Figure 1:**
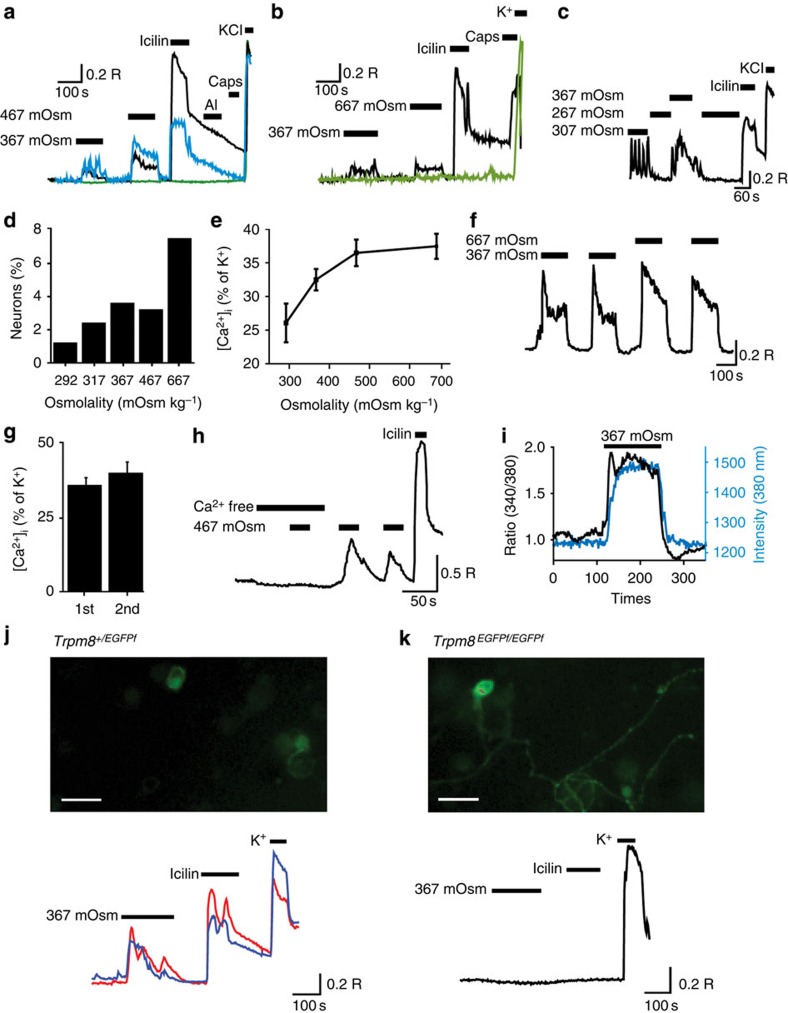
Sensory neurons are activated by hyperosmotic stimuli. (**a**) Representative examples of [Ca^2+^]_i_-responses of DRG neurons evoked by an increase in osmolality made by addition of NaCl (from 307 mOsm kg^−1^ to 367 and 467 mOsm kg^−1^). Horizontal bars indicate applications of hyperosmotic solutions, followed by icilin (1 μM), allyl isothiocyanate (AI, 100 μM), capsaicin (Caps, 1 μM) and 50 mM KCl. The fura-2 340/380 ratio (R) is measured as an index of [Ca^2+^]_i_. (**b**) Representative examples of [Ca^2+^]_i_-responses in DRG neurons evoked by an increase in osmolality made by addition of sucrose (from 267 mOsm kg^−1^ to either 367 and 667 mOsm kg^−1^) (**c**) Effect of decreasing and increasing osmolality on [Ca^2+^]_i_ fluctuations. Note spontaneous [Ca^2+^]_i_-oscillations were suppressed by reduced osmolality and enhanced by increased osmolality. (**d**) The percentages of DRG neurons responding to stimulation with different osmolalities. Osmolality was altered by changing the concentration of NaCl and neurons were perfused with a solution of 267 mOsm kg^−1^ before application of the increased osmolality. Osmolalities (mOsm kg^−1^) and numbers of responsive neurons/total number of neurons were 292: 18/1,491, 317: 31/867, 367: 81/2,240, 467: 58/867 and 667: 171/2,240. Similar percentages of neurons responded when osmolality was increased by addition of sucrose (317: 19/700 *P*>0.3, 367: 35/1,214 *P*>0.2, 467: 31/626 *P*=0.18, Fisher's exact test). *P*-values represent comparison to data obtained with NaCl. (**e**) Amplitudes of responses evoked by different osmolalities. Amplitudes expressed as a percentage of the [Ca^2+^]_i_-response evoked by a subsequent challenge with 50 mM KCl). (**f**) [Ca^2+^]_i_-responses evoked by repeated application of hyperosmotic solutions. (**g**) Mean amplitude of responses evoked by the first and second hyperosmotic challenge (increase from 307 to 367 mOsm, *n*=29 neurons, *P*=0.4, *t*-test). (**h**) Hyperosmotically evoked [Ca^2+^]_i_-responses in the absence and presence of external [Ca^2+^]_i_. Calcium-free solution contained no extracellular Ca^2+^ and 1 mM EGTA. (**i**) Traces to show time course of the Fura-2 (340/380) ratio change evoked by 367 mOsm kg^−1^ in a osmosensitive neuron (black) and the Fura-2 emission intensity (380 nm excitation) in a non-responding neuron, reflecting cell volume changes (blue). (**j**,**k**) Top: EGFPf fluorescence was used to identify *Trpm8*^*+/EGFPf*^ and *Trpm8*^*EGFPf/EGFPf*^ DRG neurons. Bottom: Fura-2 ratio changes in response to hyperosmotic solutions (NaCl addition), icilin and depolarization evoked by an additional 50 mM KCl. DRG neurons from (**j**) a heterozygous *Trpm8*^*+/EGFPf*^ mouse (scale bar, 30 μm) and (**k**) a homozygous *Trpm8*^*EGFPf/EGFPf*^ mouse (scale bar, 30 μm). The fluorescence signal in the *Trpm8*^*+/EGFPf*^ neurons was weaker than in *Trpm8*^*EGFPf/EGFPf*^ neurons.

We used [Ca^2+^]_i_-measurements to identify osmosensitive populations of neurons isolated from mouse DRG and TG. With this technique it was possible to detect the presence of mechanisms involving influx of Ca^2+^ through Ca^2+^-permeable channels as well as Ca^2+^ release from intracellular stores. Hyperosmotic challenges evoked [Ca^2+^]_i_ increases in some sensory neurons, although the majority of neurons were unresponsive (Fig. 1a). Exposure of sensory neurons to solutions made hyperosmotic by either an increase in NaCl concentration (Fig. 1a) or addition of sucrose (Fig. 1b) yielded similar results indicating that the responses were due to an increase in osmolality rather than in tonicity. Intriguingly, many of these neurons displayed oscillations in [Ca^2+^]_i_ in solutions of standard osmolality (∼310 mOsm kg^−1^), which were rapidly reduced or completely suppressed when the osmolality of the solution was decreased (Fig. 1c). When the osmolality was returned to normal levels the [Ca^2+^]_i_ oscillations reappeared, often at an augmented level and with a more sustained increase in [Ca^2+^]_i_. This reversible baseline activity suggested that an osmosensitive process operated in these neurons at normal physiological osmolalities, which in mice are in the range of 300–330 mOsm kg^−1^ (refs 2, 13, 14). We therefore examined the responses of sensory neurons to increases in osmolality starting from a slightly hypo-osmotic level of 267 mOsm kg^−1^ where the baseline activity was suppressed. Under these conditions, an increase in osmolality by 25–400 mOsm kg^−1^ by addition of NaCl (final osmolality 292–667 mOsm kg^−1^) evoked increases in [Ca^2+^]_i_ in a small percentage of sensory neurons (Fig. 1c,d). Increases in [Ca^2+^]_i_ were observed in 3.6% (81/2,240) of DRG neurons and 4.1% (35/846) of TG neurons when the osmolality was increased by 100 mOsm kg^−1^ (from 267 to 367 mOsm kg^−1^). The percentage of activated neurons increased with increasing osmolality (Fig. 1d) and the mean [Ca^2+^]_i_-response amplitude increased with increasing osmotic strength (Fig. 1e).

Since the responses evoked by hyperosmotic stimulation showed little or no desensitization (Fig. 1f,g), it was possible to investigate the response characteristics using repeated hyperosmotic challenges. The responses were dependent on a calcium entry pathway as the increase in [Ca^2+^]_i_ was completely abrogated when neurons were stimulated with a Ca^2+^-free hyperosmotic solution (Fig. 1h).

We monitored the change in cell volume by measuring the Fura-2 emission at a single wavelength (either 340 or 380 nm) in adjacent, non-osmotically responsive neurons during the challenge with hyperosmotic solutions. This approach has previously been used to monitor changes in cell volume^15^,^16^. When cells shrink, the Fura-2 dye concentrates within the cell, leading to an increase in the emitted light for both excitation wavelengths. In cells that are not osmotically activated, this is not accompanied by any change in the 340/380 Fura-2 emission ratio, whereas osmotically activated neurons show a change in the 340/380 ratio. The osmotically evoked changes in [Ca^2+^]_i_ in activated neurons occurred rapidly after increasing and decreasing extracellular osmolality and closely followed the time course of the changes in cellular volume measured in adjacent non-responsive neurons (Fig. 1i). The osmotically evoked changes in neuronal volume could also be visualized directly by the changes in cell size in the sequence of Fura-2 ratio images (see Supplementary Movie 1).

### Hyperosmotically activated neurons express TRPM8

TRP channels have been implicated in osmotic responses of sensory neurons and are useful markers of different modalities of sensory neurons. We therefore challenged the neuronal preparations with a series of sensory neuron TRP channel agonists to determine any correlation between TRP channel expression and hyperosmotic responses. There was a striking correlation in the responses to hyperosmotic solutions and icilin (Fig. 1a–c,h). For example, 69.1% (56 out of 81) of DRG neurons and 86% (30 out of 35) of trigeminal neurons that responded to a mild hyperosmotic challenge with an increase in [Ca^2+^]_i_, subsequently responded to icilin (see Supplementary Movie 1). Overall, 4.7% (105 out of 2,240) of DRG neurons and 10.2% (86 out of 846) of TG neurons responded to the TRPM8 agonist icilin, similar to previously published data for TRPM8 expression in these ganglia^17^,^18^,^19^,^20^. In contrast, we found no clear correlation between responses to other TRP channel agonists (allyl isothiocyanate—AITC for TRPA1; capsaicin for TRPV1) and hyperosmotically evoked [Ca^2+^]_i_ responses (see, for example, Fig. 1a).

A striking feature of the hyperosmotically responsive neurons was their small size typical of TRPM8-expressing neurons^17^,^20^. The mean diameter of the isolated osmosensitive neurons was 11.7±0.3 μm (*n*=103), compared with that of capsaicin-responsive neurons, which are known to be of small to medium size and measured 21.1±0.7 μm (*n*=40, *P*<0.001, *t*-test). We next studied neurons cultured from homozygous (*Trpm8*^*EGFPf/EGFPf*^) mice, which express farnesylated EGFPf under the control of the TRPM8 promoter^18^. These had a mean diameter of 11.1±0.2 μm (*n*=89), consistent with the size of the isolated neurons responsive to icilin and hyperosmotic solutions, but somewhat smaller than the diameter reported for the same population of fluorescent neurons in DRG sections^18^,^19^. We then studied the osmosensitivity of identified EGFPf-expressing DRG neurons from heterozygous *Trpm8*^*EGFPf/+*^ mice by increasing the osmolality from 267 to 367 mOsm kg^−1^. The majority of hyperosmotically responsive neurons (*n*=23/29, 79%) showed a clear EGFPf flurorescence (Fig. 1j) and all 23 were icilin sensitive (Fig. 1j). In contrast, only 1.1% (6/531) of non-EGFPf-expressing neurons responded to the hyperosmotic stimulus.

**Figure 2:**
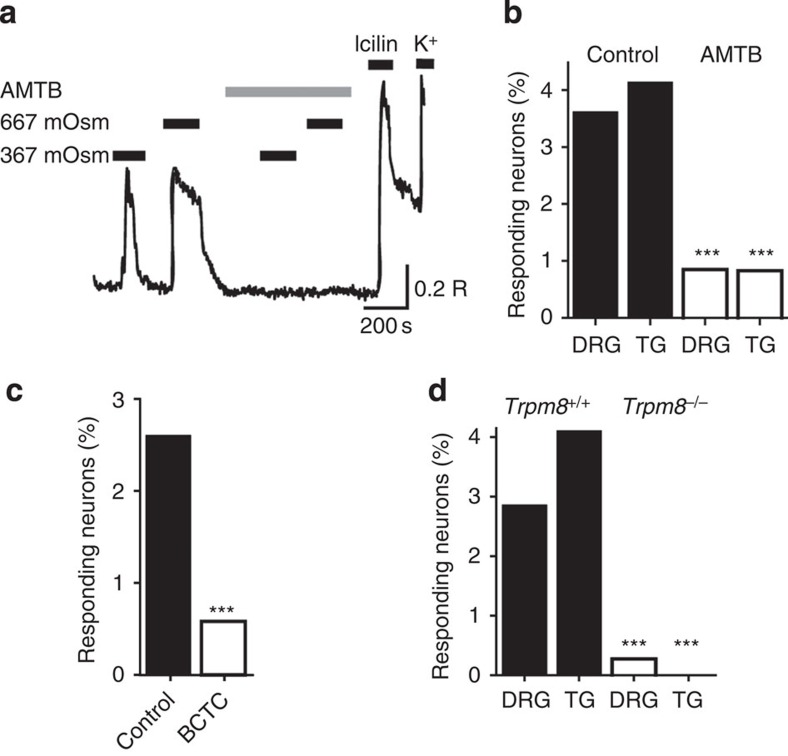
Hyperosmotically activated DRG neurons express TRPM8. (**a**) [Ca^2+^]_i_ responses monitored by Fura-2 in an icilin-sensitive neuron. Osmolalities were increased to the levels indicated from an initial value of 267 mOsm kg^−1^ by addition of NaCl except where stated. Application of the TRPM8 antagonist AMTB (30 μM) is indicated by a grey bar. Neurons were identified by a final depolarizing challenge with a solution containing 50 mM KCl. (**b**) The percentage of mouse DRG and TG neurons responding to hyperosmotic stimulation (367 mOsm kg^−1^) in the presence and absence of the TRPM8 antagonist AMTB (30 μM). AMTB reduced the percentage of hyperosmotically activated DRG neurons from 3.6% (*n*=81/2,240) to 0.9% (*n*=19/2,240) and TG neurons from 4.1% (*n*=35/846) to 0.8% (*n*=7/846). (**c**) The percentage of mouse DRG and TG neurons responding to hyperosmotic stimulation (367 mOsm kg^−1^) in the presence and absence of the TRPM8 antagonist BCTC (3 μM). BCTC reduced the percentage of DRG neurons responding to 367 mOsm kg^−1^ from 2.6% (*n*=55/2,131) to 0.6% (*n*=12/2131). (**d**) Percentage of mouse DRG and TG neurons from *Trpm8*^*+/+*^ and *Trpm8*^*−/−*^ mice responsive to a hyperosmotic stimulus (367 mOsm kg^−1^). Absence of TRPM8 reduced the percentage of hyperosmotically activated DRG neurons from 2.6% (*n*=52/1,843) to 0.3% (*n*=4/1,453) and TG neurons from 4.1% (*n*=86/846) to 0% (*n*=0/460). ***P<0.001, Fisher's exact test. In experiments where osmolality was increased to 367 mOsm kg^−1^ by addition of sucrose, the percentage of responsive DRG neurons was similarly reduced (*P*>0.2, Fisher's Exact Test) from 2.9% (*n*=35/1,214) to 0.6% (*n*=7/1,174, *P*<0.0001).

To determine whether TRPM8 played an essential role in the hyperosmotic responses or simply acted as a marker for the responsive neurons, we examined the effects of TRPM8 antagonists and deletion of functional TRPM8 channels on the sensory neuron responses. For these experiments, we took advantage of the lack of desensitization of the hyperosmotic [Ca^2+^]_i_ responses (Fig. 1f,g). In control experiments, similar sized responses were evoked by two consecutive hyperosmotic challenges in the absence of antagonists (Fig. 2a,b). Neuronal cultures were then exposed to hyperosmotic solutions first in the absence and then in the presence of either *N*-(3-aminopropyl)-2-{[(3-methylphenyl)methyl]oxy}-*N*-(2-thienylmethyl) benzamide (AMTB^21^) or N-(4-tertiarybutylphenyl)-4-(3-cholorphyridin-2-yl)tetrahydropryazine-1(2H)-carbox-amide (BCTC^22^), to determine the effect of TRPM8 inhibition on [Ca^2+^]_i_-responses evoked by increased osmolality. The concentration of AMTB used (30 μM) was sufficient to substantially inhibit TRPM8 responses to icilin or menthol (our unpublished IC_50_ value ∼3 μM). AMTB almost completely inhibited the hyperosmotic responses in over 75% of DRG neurons (*n*=62/81 responsive neurons) and 80% of TG neurons (*n*=28/35), reducing the mean response amplitude by 92% in DRG neurons and 91% in TG neurons (Fig. 2a,b). Similarly, BCTC (3 μM) essentially abolished the hyperosmotic responses in 78% of DRG neurons (43/55, Fig. 2c), and reduced the mean [Ca^2+^]_i_ response amplitude by 89%. We next examined the osmotic sensitivity of sensory neurons from *Trpm8*^*−/−*^ mice and wild-type littermates. Responses to hyperosmotic stimuli were essentially absent in neurons from *Trpm8*^*−/−*^ mice (only 4/1,453 DRG neurons and 0/460 TG neurons responded, Fig. 2d). Furthermore, none of the EGFPf-expressing neurons (0/18) from homozygous (*Trpm8*^*EGFPf/EGFPf*^) knockout mice responded to the hyperosmotic stimulus (see Fig. 1k). These two lines of evidence demonstrate that functional TRPM8 channels are necessary for sensory neuron responses to relatively mild increases in osmolality.

### Osmotic sensitivity of TRPM8

**Figure 3:**
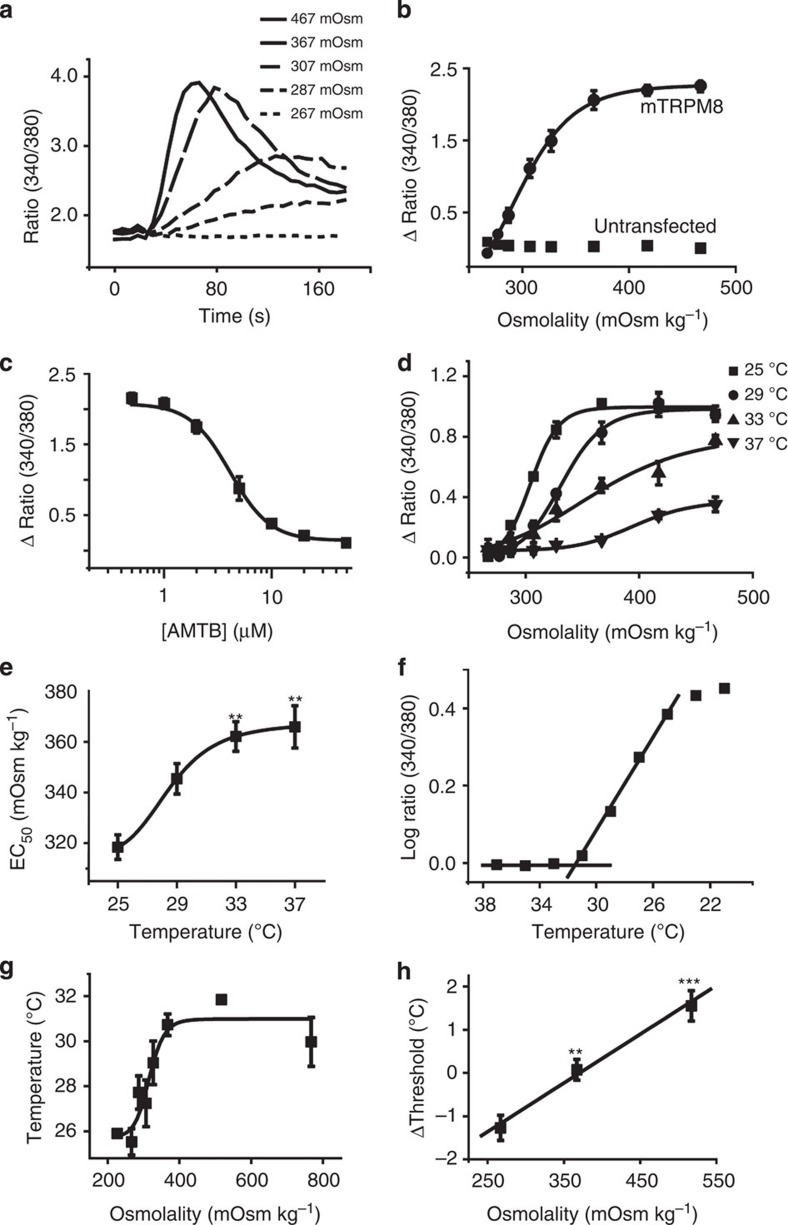
TRPM8 osmosensitivity is modulated by temperature. (**a**) Time course of [Ca^2+^]_i_ responses evoked by raising the osmolality from 267 mOsm kg^−1^ to the values shown by addition of sucrose. Solution changes at 20 s. (**b**) Osmolality-[Ca^2+^]_i_ response relationship for mTRPM8-expressing CHO cells. EC_50_ value was 318±5 mOsm kg^−1^ (*n*=9). Note the absence of evoked increase in [Ca^2+^]_i_ in untransfected CHO cells. (**c**) Activation of TRPM8 by a hyperosmotic solution (367 mOsm kg^−1^) in the presence of increasing concentrations of AMTB, IC_50_ 4±0.3 μM (*n*=3). (**d**) Osmolality-response curves at 25, 29, 33 and 37 °C (curves are representative examples of nine experiments). (**e**) EC_50_ values for osmolality evoked responses in mTRPM8 CHO cells at 25, 29, 33 and 37 °C (*n*=9). *P* values represent comparison to 25 °C, **P<0.01, Kruskal–Wallis test with pairwise comparisons. (**f**) Temperature activation thresholds were determined by plotting the log Fura-2 (340/380) ratio against temperature. The temperature at which the log ratio begins to deviate from baseline was used as the temperature activation threshold. The graph illustrates a [Ca^2+^]_i_ response evoked by a cooling ramp in a mTRPM8 CHO cell population (367 mOsm kg^−1^). (**g**) Temperature activation thresholds of mTRPM8 CHO cells at different osmolalities (*n*=4–5 experiments (minimum of 19 cells per experiment)). (**h**) Change in temperature thresholds in response to two sequential cooling ramps. First ramp in solution of 267 mOsm kg^−1^, second ramp at osmolalities indicated. Neurons exposed to an increase in osmolality exhibited shifts in activation thresholds to warmer temperatures (267 mOsm kg^−1^, *n*=38 neurons. 367 mOsm kg^−1^, *n*=85 neurons. 517 mOsm kg^−1^, *n*=55), *P* values represent comparison to 267 mOsm kg^−1^. **P<0.01, ***P<0.001, Kruskal–Wallis, followed by Dunn-Bonferroni's pairwise *post-hoc* test. Increases in osmolality in experiments (**a**–**h**) were made by addition of sucrose.

To determine if TRPM8 was sufficient to confer osmosensitivity or if a sensory neuron environment was essential for hyperosmotic responses, we examined the responses of CHO cells heterologously expressing mouse TRPM8. Here we discovered that TRPM8 CHO cells were highly sensitive to small changes in osmolality, and like some of the sensory neurons showed a degree of constitutive activity at normal physiological osmolalities (307 mOsm kg^−1^). To eliminate the baseline constitutive activity, we therefore used a starting solution with slightly reduced osmolality (267 mOsm kg^−1^) to study the relationship between osmolality and evoked increases in [Ca^2+^]_i_. Raising the osmolality by addition of sucrose evoked concentration-dependent increases in [Ca^2+^]_i_ that reached a plateau level at final osmolalities above 367 mOsm kg^−1^ (Fig. 3a). The sensitivity to increases in osmolality was independent of the agent added to the extracellular solution, with similar responses and EC_50_ values seen with addition of either NaCl or sucrose (Supplementary Fig. 1a, EC_50_ values: sucrose=318±5 mOsm kg^−1^, *n*=9; NaCl=313±8 mOsm kg^−1^, *n*=5, *P*>0.5, *t*-test). The sensitivity to increased osmolality was not limited to mouse TRPM8, as we observed a similar osmosensitivity in CHO cells expressing human TRPM8 (Supplementary Fig. 1b; EC_50_=291±16 mOsm kg^−1^, *n*=3, *P*>0.05, *t*-test). Our results demonstrate that TRPM8 was essential for the osmotic TRPM8 CHO cell responses. First, no [Ca^2+^]_i_ responses were evoked in untransfected CHO cells by hyperosmotic solutions over the range that activated TRPM8 CHO cells (Fig. 3b). Second, AMTB inhibited the hyperosmotic responses of TRPM8 CHO cells in a concentration-dependent manner with an IC_50_ value (4.0±0.3 μM, *n*=3) similar to that required to inhibit menthol- or icilin-evoked responses (Fig. 3c).

### Interactions between cold and osmolality

As TRPM8 is a thermosensitive channel, we investigated the influence of temperature on osmotic responses. [Ca^2+^]_i_-responses of TRPM8 CHO cells to changes in osmolality were determined at different temperatures and the EC_50_ values plotted against temperature. A clear temperature sensitivity was noted as the EC_50_ for activation increased and the amplitude of the response decreased as the temperature was raised towards 37 °C (Fig. 3d,e). Next, we examined the effects of osmolality on the temperature threshold for TRPM8 activation (Fig. 3f). In an extracellular solution with an osmolality of 307 mOsm kg^−1^, TRPM8 CHO cells responded to a cooling ramp with an increase in [Ca^2+^]_i_ at a threshold temperature of 27.2 ±1.0 °C, (*n*=4) in line with previous observations^17^,^20^. Reducing the osmolality of the external solution lowered the threshold temperature (*n*=4 independent experiments), whereas increasing osmolality elevated the threshold temperature to a plateau level at ∼31 °C (Fig. 3g). Increasing osmolality therefore raised the threshold for TRPM8 activation towards more physiologically relevant temperatures.

This interaction between osmolality and temperature thresholds for cold activation was also seen in sensory neurons. Here temperature thresholds were measured in response to two consecutive cooling ramps. Under control conditions, without a change in osmolality, we noted that the threshold for cold activation was shifted to lower temperatures for the second cold ramp, suggesting some degree of desensitization. Nevertheless, we noted a shift in threshold to warmer temperatures when the osmolality was increased for the second challenge and a greater decrease in temperature threshold in hypo-osmotic solutions (Fig. 3h).

### Other TRP channels are not activated by hyperosmolality

Sensory neurons express other TRP channels (TRPV1, TRPV2, TRPA1, TRPM3, TRPC3/6, TRPC5 and TRPC1). To determine whether other thermosensitive TRP channels respond to hyperosmolality, we performed experiments on CHO cells expressing TRPA1, TRPV1, TRPV3 or TRPM3. Increasing the osmolality up to ∼467 mOsm kg^−1^ failed to evoke any significant increases in [Ca^2+^]_i_ in these cell lines (Supplementary Fig. 2). TRPV4 is an osmotically activated ion channel that responds to reduced osmolality^23^,^24^ and has been linked behaviourally to responses to hyperosmolality in inflammatory conditions^25^. We therefore studied responses in human embryonic kidney (HEK) cells heterologously expressing human TRPV4. Although GSK1016790A, a specific TRPV4 agonist^26^, evoked concentration-dependent increases in [Ca^2+^]_i_ in these cells, raising the osmolality failed to evoke any [Ca^2+^]_i_ increase. (Supplementary Fig. 2). Furthermore, in cultured mouse DRG neurons, the TRPV4 agonist GSK1016790A (200 nM) only elicited [Ca^2+^]_i_-responses in a few DRG neurons (0.5%, *n*=5/950 neurons), none of which responded to hyperosmotic stimuli. These findings demonstrate that TRPV4 does not contribute to sensory neuron responses to hyperosmolality.

The marked reduction in the number of neurons responding to hyperosmotic solutions when TRPM8 was either inhibited pharmacologically or genetically ablated demonstrates that TRPM8 is the primary ion channel mediating neuronal responses to modest increases in osmolality at physiologically relevant levels.

### Hyperosmotically activated membrane currents

**Figure 4:**
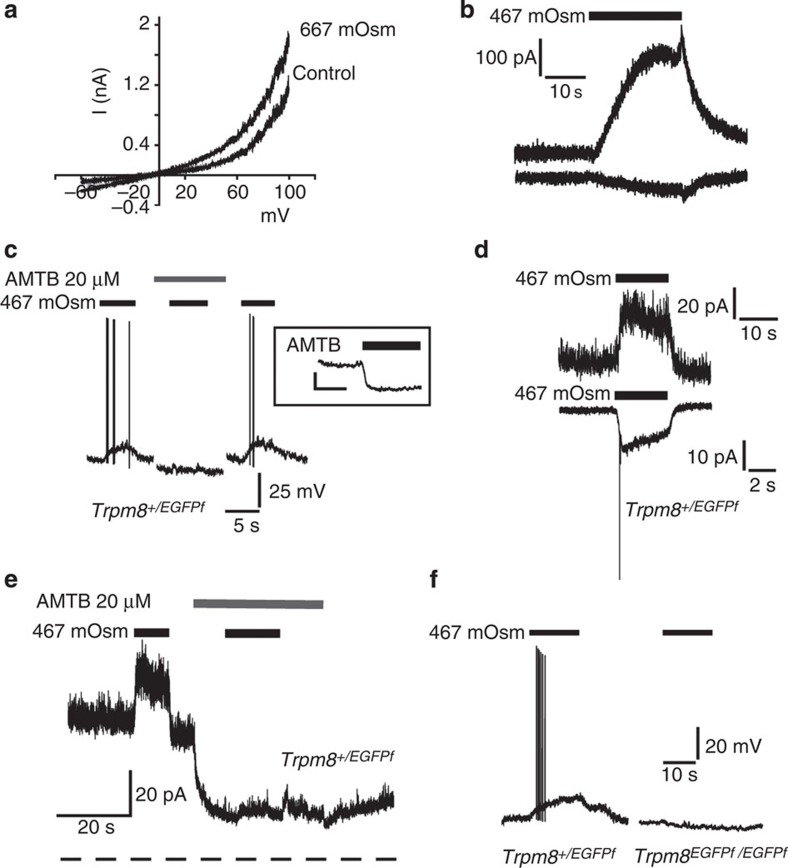
Hyperosmotic stimuli evoke membrane currents in heterologously expressing mouse TRPM8 CHO cells and Trpm8^+/EGFPf^ DRG neurons. The osmolality was altered by addition of sucrose to keep the ionic gradients constant in **a**–**f**. (**a**) Representative current responses evoked by depolarizing voltage ramps in CHO cells expressing TRPM8 channels. Voltage ramps (2 s duration) from −60 to +100 mV in 317 mOsm kg^−1^ and hyperosmotic (667 mOsm kg^−1^) extracellular solutions. (**b**) Osmotically activated outward (top trace, +60 mV) and inward (bottom trace, −60 mV) currents in a TRPM8-expressing CHO cell. Horizontal bars indicate application of hyperosmotic solutions. (**c**) Voltage (current clamp) recording demonstrating membrane depolarization and action potential firing evoked by hyperosmotic solutions in EGFP-positive neurons from a *Trpm8*^*+/EGFPf*^ mouse and the effect of the TRPM8 antagonist AMTB (20 μM). Note that application of AMTB evoked hyperpolarizations in TRPM8-expressing neurons (inset box, scale bars indicate 10 mV and 10 s). (**d**) Outward (top trace, +60 mV) and inward (bottom trace. −60 mV) currents evoked by a hyperosmotic solution (467 mOsm kg^−1^) in EGFP-positive DRG neurons from a *Trpm8*^*+/EGFPf*^ mouse. Inward current was associated with action currents from neurites in some neurons. (**e**) Outward current evoked by a hyperosmotic solution at +60 mV in a neuron from *Trpm8*^*+/EGFPf*^ mouse and the effect of AMTB. Note reduction in holding current in the presence of AMTB consistent with inhibition of voltage activated TRPM8 channels. Dotted line indicates the zero current level. (**f**) Effect of hyperosmotic solutions on membrane potential of EGFP-positive neurons from *Trpm8*^*+/EGFPf*^ mice (left) and *Trpm8*^*EGFPf /EGFPf*^ (knockout) mice (right).

We also investigated the osmotic responses of TRPM8-expressing cells and neurons electrophysiologically. In these experiments, we altered the osmolality of the external solutions by addition of sucrose, to avoid perturbing ionic gradients. TRPM8, like other sensory neuron TRP channels, shows a voltage-dependent activation that is evident at positive membrane potentials in the absence of other activating stimuli. TRPM8 agonists activate the channels, at least in part, by shifting the voltage sensitivity so that the channels open at more negative, physiologically relevant membrane potentials^27^. We therefore examined the effect of increasing osmolality on the voltage activation of TRPM8 CHO cells. As with agonists such as menthol or icilin, raising the osmolality of the external solution shifted the voltage activation to less positive potentials (Fig. 4a) and evoked outwardly rectifying currents (Fig. 4b). Stimulation of TRPM8 CHO cells with a solution of 667 mOsm kg^−1^ thus elicited small inward currents (mean current density at −60 mV, −1.24±0.23 pA/pF, *n*=7) and larger outward currents (mean current density at +60 mV, 21.30±4.91 pA/pF, *n*=9).

We examined the effects of hyperosmotic solutions on TRPM8-expressing sensory neurons. For these experiments, we studied fluorescent neurons from *EGFPf Trpm8* mice^18^. Under current clamp (voltage recording) conditions, application of hyperosmotic solutions to neurons from heterozygous *Trpm8*^*+/EGFPf*^ mice evoked a depolarization (8.96±1.31 mV, *n*=8) associated with action potential firing, which was inhibited by the TRPM8 antagonist AMTB (0.47±0.52 mV, *n*=8, *P*<0.001) and fully recovered after removal of AMTB (9.5±1.4 mV, *n*=8, *P*>0.9, ANOVA) (Fig. 4c). The depolarizing response was also absent in EGFPf-expressing neurons from *Trpm8*^*EGFPf/EGFPf*^ (TRPM8 knockout) mice (*Trpm8*^*EGFPf/EGFPf*^, −0.08±2.17 mV, *n*=10, *Trpm8*^*+/EGFPf*^ 9.98±0.98 mV, *n*=13, *P*<0.001; Fig. 4f).

Application of AMTB hyperpolarized (−11.07±1.47 mV, *n*=10) TRPM8-expressing (*Trpm8*^*+/EGFPf*^) neurons (Fig. 4c), but this was not observed in fluorescent neurons from *Trpm8*^*EGFPf/EGFPf*^ (knockout) mice (+1.00±1.37 mV, *n*=5, *P*<0.001). These findings indicate a depolarization due to ongoing, tonic TRPM8 activity consistent with the findings of constitutive TRPM8 activity at normal osmolalities in our calcium imaging studies on neurons (see Fig. 1b).

Under voltage clamp, an increase in osmolality evoked inward currents in EGFPf-positive TRPM8-expressing (*Trpm8*^*+/EGFPf*^) neurons at −60 mV and larger outward currents at positive membrane potentials (Fig. 4d). The response at negative membrane potentials was sometimes accompanied by small ‘action currents' (see Fig. 4d) indicative of action potential firing in neurites that developed during and after the first day in culture. The larger responses at positive membrane potentials facilitated investigation of the evoked currents and the role of TRPM8. The current responses typically developed within seconds of increasing osmolality, consistent with the time course noted in calcium imaging experiments. Addition of AMTB completely inhibited the hyperosmotically activated outward currents in TRPM8-expressing (*Trpm8*^*+/EGFPf*^) DRG neurons and also reduced the outward holding current (Fig. 4e). Next, we compared the responses to hyperosmotic stimulation in voltage-clamped neurons from *Trpm8*^*EGFPf/EGFPf*^ (knockout) and *Trpm8*^*+/EGFPf*^ mice. In TRPM8-expressing heterozygous mice, all EGFP-positive neurons studied responded with an outward current (mean current density at +80 mV, 5.05±0.46 pa/pF, *n*=8). In contrast, none of the EGFP-positive neurons from knockout *Trpm8*^*EGFPf/EGFPf*^ mice responded to the hyperosmotic stimulus with a significant increase in current (0.40±0.21 pA/pF, *n*=8, *P*<0.001).

Overall, the data from calcium imaging and electrophysiology experiments indicate a compulsory role for TRPM8 as a transduction molecule for hyperosmotic stimuli in isolated sensory neurons.

### Hyperosmotic solutions excite corneal nerve terminals

**Figure 5:**
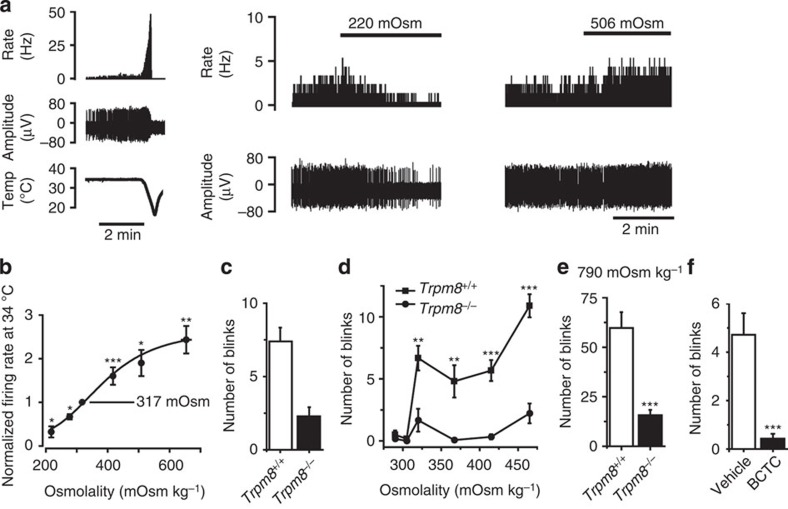
Osmolality and TRPM8 regulate corneal nerve terminal activity and blinking. (**a**) *Left hand panel:* nerve terminal impulse (NTI) activity in a cold-sensitive corneal nerve terminal exposed to a cold ramp. The traces show the firing rate (top), the NTI activity (middle) and the temperature (Temp; bottom). Cold-sensitive terminals display constitutive impulse activity, which is strongly increased in response to cooling. *Right hand panel*: The effect of reduced and increased osmolality on mean firing rate (top) and impulse activity (bottom). (**b**) Relationship between NTI activity in cold-sensitive nerve terminals (recorded at 34 °C) and external osmolality (normalized to the firing rate of each individual terminal at 317 mOsm kg^−1^, *n*=4–7). *P* values represent comparison to 317 mOsm kg^−1^ (paired *t*-test). The osmolality was altered by supplementing a physiological saline solution with sucrose to keep ionic concentrations constant. (**c**–**f**) Number of blinks measured over 2 min observation periods. Solutions of the indicated osmolalities were prepared by supplementing PBS (286 mOsm kg^−1^) with NaCl. (**c**) The number of blinks measured in untreated *Trpm8*^*−/−*^ and *Trpm8*^*+/+*^ mice (*t*-test). (**d**) The number of blinks evoked by instillation of solutions of different osmolalities in *Trpm8*^*−/−*^ *and Trpm8*^*+/+*^ mice (*t*-test). *P* values represent comparison to *Trpm8*^*−/−*^ mice. (**e**) Blinking evoked by a high osmolality (noxious) stimulus (790 mOsm kg^−1^) in *Trpm8*^*−/−*^ and *Trpm8*^*+/+*^ mice (*t*-test). (**f**) Blinking rate evoked by instillation of 465 mOsm kg^−1^ in wild-type mice treated with vehicle or BCTC (20 μM, 10 μl, *n*=7, *t*-test). For all panels, analysis of variance followed by Tukey's HSD test except where indicated. *P<0.05, **P<0.01, ***P<0.001.

In a final set of *in vitro* experiments, we extended the studies to a corneal preparation, which allowed us to examine the effects of osmolality on NTI activity in TRPM8-expressing fibres. In this preparation, neuronal activity in the terminals can be recorded by a microelectrode applied to the corneal surface. Cold-sensitive TRPM8-expressing fibres in mouse cornea can readily be identified by their spontaneous firing, which depends on TRPM8 activity and is completely absent in *Trpm8*^*−/−*^ mice^28^. The effects of altering osmolality at a controlled temperature (33 °C) are illustrated in Fig. 5a,b. Increasing the osmolality by addition of sucrose increased the spontaneous firing rate, whereas application of hypo-osmotic solutions reduced the firing rate (Fig. 5a). The osmolality–response relationship for the firing of these TRPM8-expressing corneal afferents (Fig. 5b) was similar to that observed for the osmotic sensitivity of TRPM8 expressed in CHO cells.

### Role of TRPM8 in ocular responses

Finally, we investigated the physiological effects of applying solutions of different osmolalities to the eyes of *Trpm8*^*−/−*^ and wild-type mice. A striking observation was that eye blinking was greatly reduced in untreated *Trpm8*^*−/−*^ mice compared with wild-type controls (Fig. 5c). We therefore investigated the number of blinks observed after application of solutions of different osmolality to the eyes of wild-type and *Trpm8*^*−/−*^ mice (Fig. 5d). Solutions of different osmolalities were prepared by supplementing phosphate-buffered saline with NaCl. Administration of a hypo-osmotic solution (PBS, 286 mOsm  kg^−1^) to the eyes of wild-type mice greatly reduced the blink rate from that seen in untreated mice, whereas the number of blinks was maintained at the normal level when the osmolality was at physiological levels (∼320 mOsm kg^−1^). Increasing the osmolality above this level (up to 465 mOsm kg^−1^) had no major effect on the blink rate. However, the blink frequency was significantly increased in response to application of much higher osmolalities (790 mOsm  kg^−1^; Fig. 5e). Critically, the eye blink rate was dramatically reduced in *Trpm8*^*−/−*^ mice at all osmolalities tested, including physiological osmolality (Fig. 5d). The reduction in blinking in *Trpm8*^*−/−*^ mice was also evident at the highest (noxious) osmolality tested (790 mOsm kg^−1^) where the number of blinks was dramatically increased by the hyperosmotic solution in wild-type mice but only modestly raised in *Trpm8*^*−/−*^ mice (Fig. 5e).

As it was possible that the reduced blinking frequency in the *Trpm8*^*−/−*^ mice could be explained by TRPM8 expressed at other sites than in the corneal afferent fibres, we examined the effect of topical application of a TRPM8 antagonist in wild-type mice. Local application of the TRPM8 antagonist BCTC for 10 min significantly reduced the number of eye blinks compared with that seen in mice not pre-treated with BCTC (Fig. 5f). Local inhibition of TRPM8 in corneal fibres was thus sufficient to suppress blinking.

We used a thermal imaging camera to monitor thermal emissivity as an index of corneal surface temperature during normal blinking in mice to determine whether temperature fluctuations are likely to trigger blinking. The thermal emissivity along a linear profile across the eyes of wild-type and *Trpm8*^*−/−*^ mice was stable before and after blinking (Supplementary Fig. 3a,b). The mean temperature in the centre of the eye remained unchanged from immediately before to just after blinking (Supplementary Fig. 3c, Δtemperature emissivity in *Trpm8*^*+/+*^=−0.01±0.04 °C, *n*=5, *P*>0.05, *t*-test; in *Trpm8*^*−/−*^=−0.01±0.03 °C, *n*=5 *P*>0.05, *t*-test). Blinking is thus not initiated by cooling of the corneal surface between eye blinks in mice. We also measured the corneal thermal emissivity in human volunteers. The corneal surface temperature in the centre of the eye remained stable during blinking, with the blinking clearly indicated by a sudden fluctuation in thermal emissivity due to the movement of the eyelids and the cooler eyelashes (Supplementary Fig. 3d). Similar to our observations in mice, the corneal temperature in human subjects was identical before and after normal blinking (Supplementary Fig. 3d–f, Δtemperature emissivity 0.03±0.02 °C, *P*>0.05, *t*-test).

## Discussion

TRPM8 is well known as a sensory neuron cold-activated ion channel that is responsible for detecting cool and cold temperatures^29^,^30^,^31^. Our studies have demonstrated for the first time that TRPM8 also acts as an osmosensor *in vivo*, in cultured sensory neurons and when heterologously expressed in CHO cells. Several lines of evidence show that TRPM8 is responsible for the neuronal sensitivity to solutions of increasing osmolality. First the neuronal responses were restricted to small diameter sensory neurons, which are the typical size for cold-sensitive fibres. Neurons responding to hyperosmotic challenges were identified as TRPM8 expressing, either by their co-sensitivity to icilin or by EGFPf expression. The neuronal hyperosmotic responses were inhibited by two structurally unrelated TRPM8 antagonists (AMTB and BCTC), and were absent in sensory neurons from *Trpm8*^*−/−*^ mice, demonstrating that TRPM8 mediated the observed responses. Second, heterologous expression of TRPM8 was sufficient to confer sensitivity to hyperosmotic challenges to CHO cells. Third, the tonic, TRPM8-dependent impulse activity of corneal cold-sensitive nerve terminals was inhibited by reduced osmolality and enhanced by increased osmolality. Similar responses were observed in both TRPM8 CHO cells and TRPM8-expressing neurons when osmolality was increased by addition of either NaCl or sucrose demonstrating that the responses were not due to an increase in tonicity or a response to a sugar.

Importantly, TRPM8 is active at normal physiological osmolalities and is modulated by small deviations around the normal osmolality of extracellular fluids, which makes it an excellent sensor for small, physiologically relevant changes in osmolality. Hyperosmotic conditions increase the temperature threshold for thermal activation of heterologously expressed TRPM8 and of TRPM8-expressing sensory neurons, moving it closer to the normal body temperatures for skin and cornea. For example, in TRPM8 CHO cells, increased osmolality elevated the temperature threshold for cold activation to about 31 °C, which is close to estimates of the normal temperature of skin^32^, a tissue richly innervated by TRPM8-expressing sensory nerve fibres^18^,^19^. Conversely, a reduction in osmolality lowered the threshold temperature for TRPM8. TRPM8 therefore acts as a multimodal ion channel sensing both temperature and external osmolality. This property is not restricted to isolated cells as the characteristic, tonic impulse activity of TRPM8-expressing nerve terminals in the mouse cornea is enhanced by increases and inhibited by reductions in osmolality over the same range of osmolalities that modulate TRPM8 expressed in CHO cells.

Although we show that TRPM8 is necessary for the sensory neuron responses to hyperosmotic solutions, not all hyperosmotically sensitive neurons responded to icilin (∼70–86%). This may be due to the variable latency and oscillating [Ca^2+^]_i_-responses typically evoked by icilin leading to a failure to identify icilin-responsive TRPM8-expressing neurons. In addition, the TRPM8 responses to icilin are prone to desensitization^33^ and this may also have contributed to false-negative identification of TRPM8 neurons. The finding that the responses evoked by moderate hyperosmotic stimuli were absent in sensory neuron preparations from *Trpm8*^*−/−*^ mice and from EGFPf-expressing neurons from *Trpm8*^*EGFPf/EGFPf*^ (knockout) mice, and almost abrogated by TRPM8 antagonists, argues that TRPM8 activation is responsible for these responses. However, other mechanisms may operate in polymodal nociceptive neurons to mediate responses to much stronger, noxious hyperosmotic stimuli^34^.

TRPA1 and TRPV1 have been reported to be sensitive to hyperosmotic solutions. However, we found that TRPV1-expressing CHO cells and the overwhelming majority of the capsaicin-sensitive, TRPV1-expressing neurons did not respond to increases in osmolality. An N-terminal variant of TRPV1 has been implicated in the response of central osmosensory neurons to increased osmolarity^8^. We would not have identified any neurons that expressed this TRPV1 variant as it is not activated by capsaicin^35^. However, N-terminal TRPV1 variants do not to respond to hyperosmotic stimulation^36^, but act as negative regulators of TRPV1 (refs 36, 37) inhibiting responses evoked by pH, heat and capsaicin. An earlier study also reported that TRPA1-expressing neurons could be activated by hyperosmotic stimulation^38^. The absence of hyperosmotically evoked responses in DRG and TG neurons from *Trpm8*^*−/−*^ mice clearly demonstrates that TRPA1 does not contribute to the responses observed here. Furthermore, TRPA1 heterologously expressed in CHO cells failed to respond to a hyperosmotic challenge (up to ∼467 mOsm kg^−1^).

TRPV4 expressed by various cell types has been shown to be activated by hypo-osmotic stimulation^24^ and inhibited by hyperosmotic solutions^39^, but paradoxically TRPV4 has been linked to sensitivity to hyperosmotic stimulation *in vivo* after sensitization by PGE_2_ (ref. 25; but not under naïve conditions). However, this is not due to direct activation of TRPV4-expressing sensory neurons as the responses to hyperosmotic solutions reported in that study were similar in size and frequency in DRG neurons isolated from wild-type and *Trpv4*^*−/−*^ mice^25^. These published findings agree well with our data showing that TRPV4-expressing CHO and HEK293 cells did not respond to increased osmolality, and that the DRG neurons activated by the selective TRPV4 agonist GSK1016790A did not respond to hyperosmotic stimulation.

The majority of neurons innervating the cornea are polymodal nociceptors, which are responsive to mechanical insults, heat, exogenous irritant chemicals and endogenous agents that are released from immune and damaged cells in inflammatory conditions and after injury. Activation of these nerve fibres gives rise to the sensation of pain and discomfort and evokes lacrimation, increased blinking and conjunctival vasodilatation^40^. The cornea is also innervated by a smaller population of cold-sensitive thermoreceptive neurons. Although these cold-sensitive neurons are capable of sensing small changes in temperature it is not thought that this is used physiologically to monitor the temperature of the environment^41^. Evaporative cooling in the period between blinks could reduce the temperature from the baseline level^42^, but our measurements in mice and human subjects demonstrate that the corneal temperature does not change significantly during the blinking process.

Humans blink every 4–6 s to distribute ocular secretions across the cornea, a process which is vital for maintenance of ocular health, and the characteristics of rodent and human blinking are qualitatively similar^43^. The rate of blinking is determined by the activity of a central endogenous generator that is modulated by corneal afferent nerve input. Afferent activity is important for setting the basal rate of blinking as well as the increased blink rate in response to corneal stimulation^43^. Our data implicate cold thermoreceptors and TRPM8 as the neurons and ion channel responsible for the peripheral neural input from the cornea that governs the basal blink rate. Notably, blinking was greatly reduced in mice lacking functional TRPM8 channels and inhibited by topical administration of the TRPM8 antagonist BCTC to the cornea of wild-type mice. Furthermore, we show that TRPM8 is already activated at normal physiological osmolalities in cell lines, cultured neurons and in corneal afferents, and that decreases in osmolality, which inhibit TRPM8 activity, reduced the electrical activity of cold-sensitive corneal afferents and inhibited eye blinking. Small increases in osmolality in the solution applied to the cornea resulted in only a small increase in corneal afferent firing, which did not raise the blink rate. This suggests that although TRPM8-mediated corneal afferent firing is important for blinking there is a nonlinear relationship between cold-sensitive afferent firing and blink rate. However, the blink rate was significantly increased above normal when the osmolality was increased to 790 mOsm kg^−1^. Notably, blinking was greatly reduced in *Trpm8*^*−/−*^ mice at all osmolalities tested, not only at near normal osmolalities but also at the highest osmolality tested (790 mOsm kg^−1^) where polymodal nociceptive neurons are also likely to be recruited^34^, which demonstrates a broader importance of functional TRPM8 for blinking. From these observations we conclude that TRPM8 is a major sensory neuron transducer for osmotic stimuli and that TRPM8 activity in corneal nerve fibres is required to maintain normal blinking. Basal blinking is critical for the maintenance of tear film integrity and the rate is significantly elevated in dry eye patients^44^. A plausible role for these cold-sensitive neurons is therefore to sense changes in the wetness of the ocular surface.

In summary, our results demonstrate that TRPM8 is a key osmosensitive ion channel expressed by cold-sensitive neurons and point to a hitherto unsuspected role of TRPM8-expressing neurons in regulating wetness of the ocular surface by modulating the blink rate.

## Methods

### Cell culture

DRG and TG neurons were prepared from adult male or female mice. Animals were killed by cervical dislocation, as approved by the United Kingdom Home Office, and spinal ganglia were removed from all levels of the spinal cord using aseptic methods. Ganglia were incubated in 0.25% collagenase in serum-free minimum essential medium (MEM; Invitrogen) containing 1% penicillin and streptomycin for 3 h at 37 °C in a humidified incubator gassed with 5% CO_2_ in air. This was followed by 20 min incubation with 0.25% trypsin in MEM. The ganglia were then dissociated mechanically via trituration with flame polished Pasteur pipettes to obtain a suspension of single cells. Trypsin was removed by addition of 10 ml MEM (containing 10% fetal bovine serum (FBS)) followed by centrifugation at ∼168*g* (1,000 revolutions per min) for 10 min. The pellet, containing the ganglia, was re-suspended in MEM containing 1% penicillin and streptomycin, 10% FBS and 0.05% DNase. The cell suspension was then centrifuged through a 2-ml cushion of sterile 15% bovine albumin in MEM at ∼168*g* (1,000 revolutions per min) for 10 min. The pellet, containing the neurons, was then re-suspended in an appropriate volume of MEM containing 10% FBS, 50 ng ml^−1^ NGF and 10 μM cytosine arabinoside to prevent/reduce the growth of non-neuronal cells.

Isolated neurons were plated on poly-D-lysine-coated coverslips and maintained at 37 °C in an atmosphere of 95% air-5% CO_2_ in MEM AQ (Sigma) supplemented with 10% fetal bovine serum, 100 U ml^−1^ penicillin, 100 μg ml^−1^ streptomycin and 50 ng ml^−1^ of NGF (Promega) for up to 72 h before experimentation. TRPM8-null mice and wild-type littermates were bred from heterozygous mice kindly provided by Dr David Julius. EGFPf-TRPM8 mice were kindly provided by Dr Ardem Patapoutian and C57Bl6/J mice were obtained from Harlan laboratories.

Untransfected CHO cells were grown in MEM AQ supplemented with penicillin (100 U ml^−1^), streptomycin (100 μg ml^−1^) and FBS (10%). CHO cells expressing mouse or human TRPM8, mouse TRPV3 or mouse TRPA1 were grown in the additional presence of hygromycin (200 μg ml^−1^). HEK cells transiently transfected with human TRPV4 were grown in DMEM supplemented with penicillin (100 U ml^−1^), streptomycin (100 μg ml^−1^) and FBS (10%). TRPA1 expression was under the control of a tetracycline inducible promoter to avoid cell loss due to constitutive TRPA1 activity and expression was induced by addition of tetracycline 12–18 h before experimentation. CHO cells expressing mouse TRPM3 or rat TRPV1 were grown in MEM AQ supplemented with penicillin (100 U ml^−1^), streptomycin (100 μg ml^−1^), FBS (10%) and G418 (0.5 mg ml^−1^).

### Imaging changes in intracellular calcium levels

CHO cells, DRG and trigeminal neurons were loaded with 2.5 μM Fura-2 AM (Molecular Probes) in the presence of 1 mM probenecid for ∼1 h in a solution containing (in mM) 140 NaCl, 5 KCl, 10 glucose, 10 HEPES, 2 CaCl_2_ and 1 MgCl_2_, buffered to pH 7.4 (NaOH). Solutions of different osmolality, TRP channel agonists or antagonists were applied to cells by local microperfusion of solution through a fine tube placed very close to the cells being studied. The temperature of the superfused solution was regulated by a temperature controller (Marlow Industries) attached to a Peltier device with the temperature measured at the orifice of the inflow tube.

Images of a group of cells were captured every 2 s at 340 and 380 nm excitation wavelengths with emission measured at >520 nm with a microscope-based imaging system (PTI). Analyses of emission intensity ratios at 340 nm/380 nm excitation (R, in individual cells) were performed with the ImageMaster suite of software. Unless otherwise stated the illustrated neuronal responses and analyses are from experiments where osmolality was increased by addition of NaCl. Responses evoked by raising osmolality with sucrose were identical in time course, amplitude and frequency to those evoked by NaCl in neurons from wild-type mice and were similarly abrogated in neurons from *Trpm8*^*−/−*^ mice.

For experiments on neurons from *Trpm8*^*+/EGFPf*^ or *Trpm8*^*EGFPf/EGFPf*^ mice, EGFPf-expressing neurons were first located and identified by excitation at 485 nm using a dichroic mirror with a cutoff at 500 nm and a bandpass emission filter (510–560 nm). An excitation scan (450–490 nm) was also used to confirm the presence of EGFPf fluorescence in individual neurons. The filter set was then changed to a Fura-2 compatible combination (dichroic mirror, 505 nm; long pass emission filter 520 nm) for measurements of intracellular calcium responses.

Neurons were defined as displaying a hyperosmotic-induced response, if during exposure to the hyperosmotic stimulus, they exhibited a rapid increase in Fura-2 ratio, which was reversed upon the return to a baseline osmolality. All neurons were challenged with hyperosmotic solutions twice, and only neurons that displayed a repeatable response to a second hyperosmotic challenge were included. All included neurons responded with a [Ca^2+^]_i_-increase of at least 8% of the maximal amplitude produced by a subsequent challenge with 50 mM KCl.

### Solutions

A solution containing (in mM) 140 NaCl, 5 KCl, 10 glucose, 10 HEPES, 2 CaCl_2_ and 1 MgCl_2_, buffered to pH 7.4 with NaOH, was used for the initial, pilot imaging study experiments. This solution (osmolality 307–310 mOsm kg^−1^) was used to provide continuity with our previous publications studying electrophysiological and intracellular calcium concentration responses in cultured cells and sensory neurons and has an osmolality within the range of measured normal osmolalities for mouse plasma (300–330 mOsm kg^−1^)^2^,^13^,^14^. This solution was replaced in some later experiments by a modified solution of lower osmolality when we discovered that there was a low level of baseline TRPM8 activity at ∼307–310 mOsm kg^−1^. The basic reduced osmolality solution contained (in mM) 120 NaCl, 5 KCl, 10 glucose, 10 HEPES, 2 CaCl_2_, and 1 MgCl_2_, buffered to pH 7.4 (NaOH) and had a measured osmolality of 267 mOsm kg^−1^. The osmolality of this solution was then varied by addition of either sucrose or NaCl. Calcium-free solutions were made by omitting CaCl_2_ and adding 1 mM EGTA. Osmolalities were measured by freezing point depression using an osmometer (Roebling 13).

### 96-Well plate intracellular calcium concentration assays

Changes in intracellular calcium concentration, [Ca^2+^]_i_, were also measured in TRP channel expressing CHO cells grown in 96-well black-walled plates (Costar) using a Flexstation 3 (Molecular Devices). In these experiments, osmolality was increased by addition of sucrose, unless otherwise stated. Cells were loaded with Fura 2-AM and assays were carried out at 25 °C except where the effects of temperature were studied as indicated in the text. Basal emission ratios (340 nm/380 nm) were measured and then changes in dye emission ratio determined at various times after compound addition. Experiments were performed in triplicate wells.

### Electrophysiology

CHO cell lines and neurons were studied under voltage-clamp or current-clamp conditions using an Axopatch 200B amplifier and pClamp 10.0 software (Molecular Devices). Borosilicate glass pipettes (3–6 MΩ, 75–80% series resistance compensation) were filled with (in mM) 140 KCl or CsCl, 1 CaCl_2_, 2 MgATP, 10 EGTA and 10 HEPES. Sensory neurons were studied under voltage clamp using the CsCl-based internal solution (mM: 140 CsCl, 1 CaCl_2_, 2 MgATP, 10 EGTA and 10 HEPES) to block potassium currents. Sensory neuron current-clamp recordings were performed using an internal solution containing 135 KCl, 5 NaCl, 1 CaCl_2_, 2 MgATP, 10 EGTA and 10 HEPES. The voltage sensitivity of membrane currents were investigated using a voltage ramp protocol. The rate of voltage change during the ramp (160 mV over 2 s) was sufficiently slow that the voltage-dependent TRPM8 currents were at steady state. This was confirmed by comparing the *I*–*V* relationship obtained with this protocol with the *I*–*V* relationship obtained with a voltage step protocol where steady-state currents were achieved (data not shown). A low osmolality external solution as described above for imaging of intracellular Ca^2+^ concentrations was used for some electrophysiological recordings and hyperosmotic solutions were prepared by addition of sucrose. All recordings were carried out at room temperature.

EGFPf-expressing neurons were identified for electrophysiological examination using brief irradiation with a Xenon lamp and an FITC-compatible filter set.

### Corneal nerve terminal recordings

NTI activity from mouse cornea was recorded using the methods and solutions described elsewhere^28^. The eye was excised from the mouse and secured in placed in a small recording chamber. A broken patch pipette was placed in contact with the cornea to record NTIs. The temperature of the perfusing solution was adjusted to the desired level with a Peltier device located at the entrance of the chamber, and measured at the corneal surface. A hypo-osmotic physiological saline solution with reduced NaCl concentration was supplemented with sucrose to give the osmolalities indicated, while maintaining all ionic concentrations unchanged. Differences in NTI rates at individual osmolalities were compared with the rate at 317 mOsm kg^−1^ in a pairwise manner for the unit under study to obtain a fold-change in firing rate. A solution with an osmolality of 317 mOsm kg^−1^ was used as a reference solution and osmolality as this is within the range of measured normal osmolalities in mice and has been used as the standard in previous publications with this preparation.

### Blinking

Blinking was measured either in untreated mice or after application of solutions of different osmolality (10 μl) to the corneal surface. A slightly hypo-osmotic phosphate-buffered saline solution (286 mOsm kg^−1^) was supplemented with NaCl to give the indicated osmolalities. The mice were lightly restrained so that the solution remained on the corneal surface and the number of blinks counted over a 2-min period (see Supplementary Movie 2 for an example of eye blink responses). The solutions were then removed and the mice returned to their home cages.

### Thermal imaging

The corneal thermal emissivity of human volunteers and lightly scruffed mice was recorded at 50 Hz using a FLIR T650 camera fitted with a FOL25 lens. Thermal emissivity was measured along a linear profile across the eye and in a point in the centre of the eye, immediately before and after blinking. Data were collected using FLIR ResearchIR 3.4 and exported for further analysis. Temperature was inferred from the internal thermal emissivity calibration.

### Statistics

All data are expressed as mean±s.e.m. Differences in neuron diameters, current densities and membrane potentials were analysed by Student's *t*-test or one-way analysis of variance followed by Dunnett's test for multiple comparisons. Differences in the proportions of responding neurons were analysed using a *χ*^2^ or Fisher's exact test. In the cellular assays, differences in EC_50_ values with increasing temperature and temperature thresholds with increasing osmolality were analysed by Kruskal–Wallis test with pairwise comparisons. Changes in NTI activity were compared using a paired *t*-test. Differences in tearing and blinking rate were analysed by analysis of variance followed by Tukey's honest significant difference (HSD) test or *t*-test as indicated. Corneal thermal emissivity data were analysed by paired *t*-tests. All tests were two-sided.

## Additional information

**How to cite this article:** Quallo, T. *et al*. TRPM8 is a neuronal osmosensor that regulates eye blinking in mice. *Nat. Commun.* 6:7150 doi: 10.1038/ncomms8150 (2015).

## Supplementary Material

Supplementary Information Supplementary Figures 1-3

Supplementary Movie 1 Example of the eye blink response in a wild-type mouse. Fura-2 responses of DRG neurons to a sequential challenge with a hyperosmotic solution followed by icilin and depolarization with a high KCl concentration (50mM). Note the selective activation by the hyperosmotic stimulus of an icilin-sensitive neuron. The reversible shrinkage of the neurons in response to the hyperosmotic stimulation can also be seen in both the responsive neuron and the adjacent non-responsive neurons.

Supplementary Movie 2 Fura-2 responses of DRG neurons. An example of the eye blink response in a lightly restrained wild-type mouse following application of a hyperosmotic solution (790 mosm.kg^-1^, 10µl) to the right eye and PBS (10µl) to the left eye. A solution of higher osmotic strength was used to provide a clear demonstration of this behavioral response.

## References

[b1] Gallar J., Pozo M. A., Tuckett R. P. & Belmonte C. Response of sensory units with unmyelinated fibres to mechanical, thermal and chemical stimulation of the cat's cornea. J. Physiol. 468, 609–622 (1993). 8254527 10.1113/jphysiol.1993.sp019791 PMC1143846

[b2] Lechner S. G. . The molecular and cellular identity of peripheral osmoreceptors. Neuron 69, 332–344 (2011). 21262470 10.1016/j.neuron.2010.12.028

[b3] Fox A. J., Barnes P. J. & Dray A. Stimulation of guinea-pig tracheal afferent fibres by non-isosmotic and low-chloride stimuli and the effect of frusemide. J. Physiol. 482, 179–187 (1995). 7730981 10.1113/jphysiol.1995.sp020508 PMC1157762

[b4] Hirata H. & Meng I. D. Cold-sensitive corneal afferents respond to a variety of ocular stimuli central to tear production: implications for dry eye disease. Invest. Ophthalmol. Vis. Sci. 51, 3969–3976 (2010). 20335617 10.1167/iovs.09-4744 PMC2910635

[b5] Pedersen K. E., Meeker S. N., Riccio M. M. & Undem B. J. Selective stimulation of jugular ganglion afferent neurons in guinea pig airways by hypertonic saline. J. Appl. Physiol. 84, 499–506 (1998). 9475859 10.1152/jappl.1998.84.2.499

[b6] Belmonte C., Gallar J., Pozo M. A. & Rebollo I. Excitation by irritant chemical substances of sensory afferent units in the cat's cornea. J. Physiol. 437, 709–725 (1991). 1890657 10.1113/jphysiol.1991.sp018621 PMC1180073

[b7] Vriens J. . Cell swelling, heat, and chemical agonists use distinct pathways for the activation of the cation channel TRPV4. Proc. Natl Acad. Sci. USA 101, 396–401 (2004). 14691263 10.1073/pnas.0303329101 PMC314196

[b8] Bourque C. W. Central mechanisms of osmosensation and systemic osmoregulation. Nat. Rev. Neurosci. 9, 519–531 (2008). 18509340 10.1038/nrn2400

[b9] Venkatachalam K. & Montell C. TRP channels. Annu. Rev. Biochem. 76, 387–417 (2007). 17579562 10.1146/annurev.biochem.75.103004.142819 PMC4196875

[b10] Jin M., Berrout J. & O'Neil R. G. Regulation of TRP Channels by Osmomechanical Stress In: Zhu M. X. (ed) TRP Channels (2011). 22593965

[b11] Colbert H. A., Smith T. L. & Bargmann C. I. OSM-9, a novel protein with structural similarity to channels, is required for olfaction, mechanosensation, and olfactory adaptation in Caenorhabditis elegans. J. Neurosci. 17, 8259–8269 (1997). 9334401 10.1523/JNEUROSCI.17-21-08259.1997 PMC6573730

[b12] Liu L. . Drosophila hygrosensation requires the TRP channels water witch and nanchung. Nature 450, 294–298 (2007). 17994098 10.1038/nature06223

[b13] Sharif Naeini R., Witty M. F., Seguela P. & Bourque C. W. An N-terminal variant of Trpv1 channel is required for osmosensory transduction. Nat. Neurosci. 9, 93–98 (2006). 16327782 10.1038/nn1614

[b14] Waymouth C. Osmolality of mammalian blood and of media for culture of mammalian cells. In vitro 6, 109–127 (1970). 4943053 10.1007/BF02616113

[b15] Viana F., de la Pena E., Pecson B., Schmidt R. F. & Belmonte C. Swelling-activated calcium signalling in cultured mouse primary sensory neurons. Eur. J. Neurosci. 13, 722–734 (2001). 11207807 10.1046/j.0953-816x.2000.01441.x

[b16] Alvarez-Leefmans F. J., Herrera-Perez J. J., Marquez M. S. & Blanco V. M. Simultaneous measurement of water volume and pH in single cells using BCECF and fluorescence imaging microscopy. Biophys. J. 90, 608–618 (2006). 16258035 10.1529/biophysj.105.069450 PMC1367065

[b17] Peier A. M. . A TRP channel that senses cold stimuli and menthol. Cell 108, 705–715 (2002). 11893340 10.1016/s0092-8674(02)00652-9

[b18] Dhaka A., Earley T. J., Watson J. & Patapoutian A. Visualizing cold spots: TRPM8-expressing sensory neurons and their projections. J. Neurosci. 28, 566–575 (2008). 18199758 10.1523/JNEUROSCI.3976-07.2008 PMC6670358

[b19] Takashima Y. . Diversity in the neural circuitry of cold sensing revealed by genetic axonal labeling of transient receptor potential melastatin 8 neurons. J. Neurosci. 27, 14147–14157 (2007). 18094254 10.1523/JNEUROSCI.4578-07.2007 PMC2883248

[b20] McKemy D. D., Neuhausser W. M. & Julius D. Identification of a cold receptor reveals a general role for TRP channels in thermosensation. Nature 416, 52–58 (2002). 11882888 10.1038/nature719

[b21] Lashinger E. S. . AMTB, a TRPM8 channel blocker: evidence in rats for activity in overactive bladder and painful bladder syndrome. Am. J. Physiol. Renal. Physiol. 295, F803–F810 (2008). 18562636 10.1152/ajprenal.90269.2008

[b22] Madrid R. . Contribution of TRPM8 channels to cold transduction in primary sensory neurons and peripheral nerve terminals. J. Neurosci. 26, 12512–12525 (2006). 17135413 10.1523/JNEUROSCI.3752-06.2006 PMC6674899

[b23] Liedtke W. . Vanilloid receptor-related osmotically activated channel (VR-OAC), a candidate vertebrate osmoreceptor. Cell 103, 525–535 (2000). 11081638 10.1016/s0092-8674(00)00143-4 PMC2211528

[b24] Strotmann R., Harteneck C., Nunnenmacher K., Schultz G. & Plant T. D. OTRPC4, a nonselective cation channel that confers sensitivity to extracellular osmolarity. Nat. Cell. Biol. 2, 695–702 (2000). 11025659 10.1038/35036318

[b25] Alessandri-Haber N., Joseph E., Dina O. A., Liedtke W. & Levine J. D. TRPV4 mediates pain-related behavior induced by mild hypertonic stimuli in the presence of inflammatory mediator. Pain 118, 70–79 (2005). 16213085 10.1016/j.pain.2005.07.016

[b26] Thorneloe K. S. . N-((1S)-1-{[4-((2S)-2-{[(2,4-dichlorophenyl)sulfonyl]amino}-3-hydroxypropanoyl)-1 -piperazinyl]carbonyl}-3-methylbutyl)-1-benzothiophene-2-carboxamide (GSK1016790A), a novel and potent transient receptor potential vanilloid 4 channel agonist induces urinary bladder contraction and hyperactivity: Part I. J. Pharmacol. Exp. Ther. 326, 432–442 (2008). 18499743 10.1124/jpet.108.139295

[b27] Voets T. . The principle of temperature-dependent gating in cold- and heat-sensitive TRP channels. Nature 430, 748–754 (2004). 15306801 10.1038/nature02732

[b28] Parra A. . Ocular surface wetness is regulated by TRPM8-dependent cold thermoreceptors of the cornea. Nature Med. 16, 1396–1399 (2010). 21076394 10.1038/nm.2264

[b29] Bautista D. M. . The menthol receptor TRPM8 is the principal detector of environmental cold. Nature 448, 204–208 (2007). 17538622 10.1038/nature05910

[b30] Dhaka A. . TRPM8 is required for cold sensation in mice. Neuron 54, 371–378 (2007). 17481391 10.1016/j.neuron.2007.02.024

[b31] Colburn R. W. . Attenuated cold sensitivity in TRPM8 null mice. Neuron 54, 379–386 (2007). 17481392 10.1016/j.neuron.2007.04.017

[b32] Tajino K. . Cooling-sensitive TRPM8 is thermostat of skin temperature against cooling. PLoS ONE 6, e17504 (2011). 21407809 10.1371/journal.pone.0017504 PMC3047576

[b33] Chuang H. H., Neuhausser W. M. & Julius D. The super-cooling agent icilin reveals a mechanism of coincidence detection by a temperature-sensitive TRP channel. Neuron 43, 859–869 (2004). 15363396 10.1016/j.neuron.2004.08.038

[b34] Parra A., Gonzalez-Gonzalez O., Gallar J. & Belmonte C. Tear fluid hyperosmolality increases nerve impulse activity of cold thermoreceptor endings of the cornea. Pain 155, 1481–1491 (2014). 24785271 10.1016/j.pain.2014.04.025

[b35] Lu G., Henderson D., Liu L., Reinhart P. H. & Simon S. A. TRPV1b, a functional human vanilloid receptor splice variant. Mol. Pharmacol. 67, 1119–1127 (2005). 15644492 10.1124/mol.104.009852

[b36] Eilers H., Lee S. Y., Hau C. W., Logvinova A. & Schumacher M. A. The rat vanilloid receptor splice variant VR.5'sv blocks TRPV1 activation. Neuroreport 18, 969–973 (2007). 17558279 10.1097/WNR.0b013e328165d1a2

[b37] Vos M. H. . TRPV1b overexpression negatively regulates TRPV1 responsiveness to capsaicin, heat and low pH in HEK293 cells. J. Neurochem. 99, 1088–1102 (2006). 17018028 10.1111/j.1471-4159.2006.04145.x

[b38] Zhang X. F., Chen J., Faltynek C. R., Moreland R. B. & Neelands T. R. Transient receptor potential A1 mediates an osmotically activated ion channel. Eur. J. Neurosci. 27, 605–611 (2008). 18279313 10.1111/j.1460-9568.2008.06030.x

[b39] Guler A. D. . Heat-evoked activation of the ion channel, TRPV4. J. Neurosci. 22, 6408–6414 (2002). 12151520 10.1523/JNEUROSCI.22-15-06408.2002 PMC6758176

[b40] Belmonte C., Acosta M. C. & Gallar J. Neural basis of sensation in intact and injured corneas. Exp. Eye Res. 78, 513–525 (2004). 15106930 10.1016/j.exer.2003.09.023

[b41] Belmonte C. & Gallar J. Cold thermoreceptors, unexpected players in tear production and ocular dryness sensations. Invest. Ophthalmol. Vis. Sci. 52, 3888–3892 (2011). 21632706 10.1167/iovs.09-5119

[b42] Fujishima H., Toda I., Yamada M., Sato N. & Tsubota K. Corneal temperature in patients with dry eye evaluated by infrared radiation thermometry. Br. J. Ophthalmol. 80, 29–32 (1996). 8664227 10.1136/bjo.80.1.29 PMC505379

[b43] Kaminer J., Powers A. S., Horn K. G., Hui C. & Evinger C. Characterizing the spontaneous blink generator: an animal model. J. Neurosci. 31, 11256–11267 (2011). 21813686 10.1523/JNEUROSCI.6218-10.2011 PMC3156585

[b44] Kaido M. . Dry-eye screening by using a functional visual acuity measurement system: the Osaka Study. Invest. Ophthalmol. Vis. Sci. 55, 3275–3281 (2014). 24801509 10.1167/iovs.13-13000

