# Supplementary material for: TRPM8 is a neuronal osmosensor that regulates eye blinking in mice
Source: Nat Commun. 2015 May 22;6:7150. doi: 10.1038/ncomms8150 (PMC4455064; doi:10.1038/ncomms8150)
Supplement: Supplementary Information — Supplementary Figures 1-3 [file ncomms8150-s1.pdf]

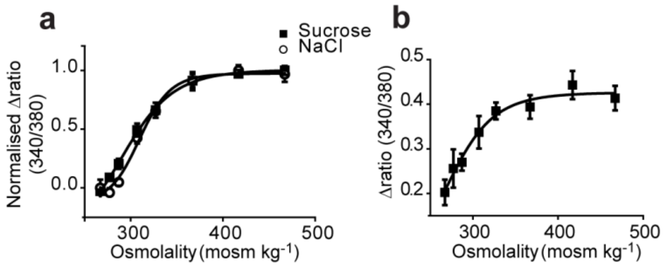

Supplementary Figure 1.

Supplementary Figure 1. ***TRPM8 is activated by increases in osmolality.*** (**a**) Increases in osmolality by addition of either sucrose or NaCl evoked similar $[Ca^{2+}]_i$ responses in Fura-2 loaded mTRPM8-expressing CHO cells. Sucrose EC_{50} - 318 ±5 mosm.kg^{-1}, n=9. NaCl EC_{50} - 313 ±8 mosm.kg^{-1}, n=5 independent experiments. (**b**) $[Ca^{2+}]_i$ responses in Fura-2 loaded, hTRPM8-expressing CHO cells evoked by solutions made hyperosmotic by addition of sucrose. EC_{50} - 291 ± 16 mosm.kg^{-1}, n=3 independent experiments.

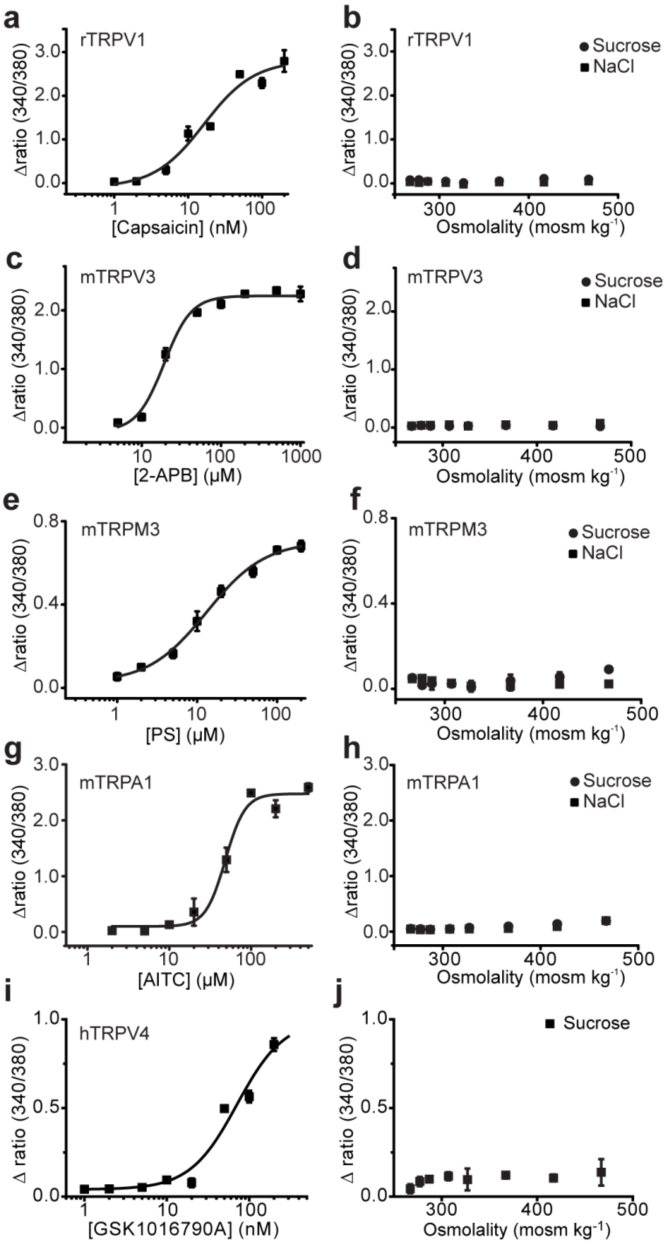

Supplementary Figure 2. ***Other sensory neuron TRP channels are not activated by hyperosmotic solutions.*** Increases in osmolality made by addition of sucrose or NaCl did not evoke $[Ca^{2+}]_i$ responses in CHO cells expressing rTRPV1 (**a,b**), mTRPV3 (**c,d**), mTRPM3 (**e,f**), mTRPA1 (**g,h**) or HEK293 cells expressing hTRPV4 (**i,j**). The results shown are representative for: rTRPV1, n=3; mTRPV3, n=3; rTRPV4 n=3; mTRPM3, n=2; mTRPA1 n=3 independent experiments. In experiments examining CHO cells stably transfected with mTRPV3 extracellular solutions containing $BaCl_2$ were used as a substitute for $CaCl_2$: here the change in Fura-2 340/380 ratio reflects the change in $[Ba^{2+}]_i$.

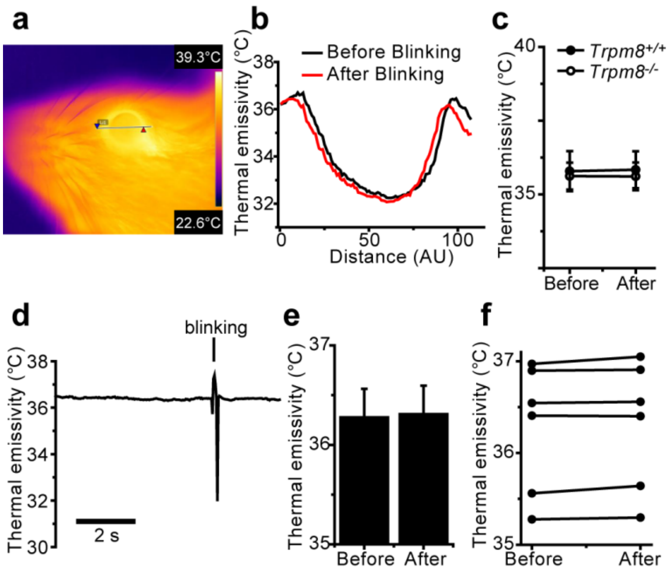

Supplementary Figure 3. ***Blinking is independent of corneal thermal emissivity.*** **(a)** Thermal emissivity from the corneal surface of mice and human subjects was measured using a thermal imaging camera. The scale bar on the right indicates the thermal emissivity as an index of surface temperature. **(b)** The thermal emissivity measured along a linear profile across the eyes of *Trpm8*$^{+/+}$ and *Trpm8*$^{-/-}$ mice was identical before and after blinking (representative trace of n=5 mice of each genotype). **(c)** Mean thermal emissivity measured immediately before and after blinking in *Trpm8*$^{+/+}$ and *Trpm8*$^{-/-}$ mice. **(d)** Time course of the corneal thermal emissivity during blinking in a human volunteer (the fluctuation indicated by “blinking” is due to eyelash movement, the trace is representative of n=6 subjects). **(e)** Mean thermal emissivity in human volunteers immediately before and after blinking. **(f)** Individual measurements of thermal emissivity before and after blinking in human subjects.
